# Plastid-Nucleus Distance Alters the Behavior of Stromules

**DOI:** 10.3389/fpls.2017.01135

**Published:** 2017-07-06

**Authors:** Jessica L. Erickson, Matthias Kantek, Martin H. Schattat

**Affiliations:** Plant Organelle Shape and Dynamics Lab, Plant Physiology, Martin Luther University Halle-WittenbergHalle, Germany

**Keywords:** stromule, retrograde signaling, nucleus movement, plastid positioning, organelle interactions, live-cell imaging, *Arabidopsis thaliana*

## Abstract

Plastids send “retrograde” signals to the nucleus to deliver information regarding their physiological status. One open question concerning this signal transfer is how the signal bridges the cytoplasm. Based on individual reports of plastid derived tubular membrane extensions connecting to nuclei, these so-called stromules have been suggested to function as communication routes between plastids and nuclei in response to biotic stress. However, based on the data currently available it is unclear whether interactions between stromules and nuclei are truly intentional or observed as a result of an inflated stromule frequency throughout the cell, and are thus a random event. The source of this uncertainty stems from missing information regarding the relative distribution of all plastids and stromules within a given cell. A comprehensive analysis of the upper epidermis of *Arabidopsis thaliana* rosette leaves was performed via a combination of still images and time-lapse movies of stromule formation in the context of the whole cell. This analysis could definitively confirm that stromule formation is not evenly distributed. Stromules are significantly more frequent within 8 μm of the nucleus, and approximately 90% of said stromules formed facing the nucleus. Time-lapse movies revealed that this enrichment of stromules is achieved via a 10-fold higher frequency of stromule initiation events within this 8 μm zone compared to the cell periphery. Following the movement of plastids and nuclei it became evident that movement and formation of stromules is correlated to nucleus movement. Observations suggest that stromules “connecting” to the nucleus are not necessarily the result of plastids sensing the nucleus and reaching out toward it, but are rather pulled out of the surface of nucleus associated plastids during opposing movement of these two organelles. This finding does not exclude the possibility that stromules could be transferring signals to the nucleus. However, this work provides support for an alternative hypothesis to explain stromule-nuclear interactions, suggesting that the main purpose of nucleus associated stromules may be to ensure a certain number of plastids maintain contact with the constantly moving nucleus.

## Introduction

It is widely accepted that plastids are descended from a free-living ancestral cyanobacteria that was enveloped by a mitochondriate eukaryote more than one billion years ago (Dyall et al., 2004). Prior to its incorporation it can be assumed that this cyanobacteria contained all coding information necessary to function autonomously as a photoautotroph (Nott et al., 2006). However, during evolution of this symbiont into the present-day chloroplast, there was a massive reduction in the size of the plastid genome, some genes being lost completely, while others were transferred to the nucleus (Dyall et al., 2004). With the reduction of the genome (contemporary plastids are know to have between 60 and 200 ORFs remaining) the chloroplast was no longer autonomous. The majority of protein coding genes necessary for chloroplast development and function in higher plants (>3,000) are now under the control of the nucleus (Nott et al., 2006). With the development of this anterograde control over the plastid it became necessary for the plastid to, in turn, communicate its physiological and developmental state to the nucleus (Nott et al., 2006). Thus, modes of so-called “retrograde signaling” between the plastid and nucleus were developed to regulate the production of nuclear encoded proteins necessary for chloroplast function and maintenance of cell homeostasis (Nott et al., 2006).

Many candidate retrograde signals have been suggested during recent years (recently summarized in Chan et al., 2016). These include carotenoid derivatives (Ramel et al., 2012; Avendaño-Vázquez et al., 2014; Van Norman et al., 2014), the isoprenoid precursor MEcPP (Xiao et al., 2012), tetrapyrrole biosynthesis derivatives (Strand et al., 2003; Woodson et al., 2011), plastid generated ROS molecules (Caplan et al., 2015; summarized in Bobik and Burch-Smith, 2015), phytohormones (Bobik and Burch-Smith, 2015), 3′-phosphoadenosine 5′-phosphate (PAP; Estavillo et al., 2011) and dual localized proteins such as PTM (Sun et al., 2011), WHIRLY1 (Isemer et al., 2012) and NRIP1 (Caplan et al., 2015). These signals can be divided into two main categories. First there are those speculated to be slower acting (e.g., plastid metabolites) because they take time to accumulate and are not capable of directly mediating changes to gene expression (Krause et al., 2012). This category of signals is likely involved in the coordination of nuclear and chloroplast gene expression during plastid development (Pogson et al., 2008). Then there is the second category of signals believed to be involved in rapid response to environmental challenges (Krause and Krupinska, 2009). In contrast to plastid metabolites, protein signals can be stored by the plastid and effectively “released” (and directly influence gene expression) when a cell requires a rapid response by the nucleus (e.g., attack by a pathogen) (Krause and Krupinska, 2009).

It is very difficult to verify the movement of signals from the plastid to the nucleus. However, notable progress has been made in the investigation of dually localized proteins (those believed to be “category 2” signals). Protein export from the plastid to the nucleus has been experimentally demonstrated on very few occasions. The first instance of experimentally detected relocation of a protein from the plastid to the nucleus was demonstrated using *Arabidopsis thaliana* WHIRLY 1 (Isemer et al., 2012). This was achieved via the generation of transplastomic tobacco plants expressing HA-tagged AtWHIRLY1. Following the exclusive synthesis of the protein by the plastid it was then identified in the nucleus, thus confirming the ability of the protein to move from plastids to the nucleus. In addition these transplastomic lines also exhibited an up-regulation of pathogen response related nuclear genes *PR1* and *PR2* (Isemer et al., 2012). A second example was the confirmed retrograde movement of the protein NRIP1 (N receptor-interacting protein 1), which was provided by Caplan et al. (2008). NRIP1 is localized to the chloroplast in unchallenged *Nicotiana benthamiana* (N-containing, NRIP1-Cerulean expressing plants), but is observed in the plastid, cytoplasm and nucleus when in complex with the Tobacco Mosaic Virus effector p50. This re-localization is further enhanced when the complex is recognized by N, the p50 immune receptor.

Retrograde protein signals, such as WHIRLY1 and NRIP1, face multiple obstacles along the road to altered nuclear gene expression. To enter the nucleus they must cross multiple lipid bilayers, the plastid outer-membrane and the nuclear membrane. In the case of NRIP1 it seems that this is unlikely to occur via simple diffusion across membranes, although the exact mechanism of nuclear entry remains unsolved (Caplan et al., 2008). A second obstacle to re-localization of a protein from the plastid to the nucleus is its movement across the cytoplasm. Several potential modes of transport have been discussed, including passive diffusion as well as active transport across the cytoplasm via as yet unidentified “transporter proteins” (Leister, 2012). In contrast, one theory gaining momentum suggests that proteins (and perhaps other putative signaling molecules) are transferred via direct contact between the plastid and the nucleus, largely suggested to occur via stromules (Caplan et al., 2008, 2015; Leister, 2012; Bobik and Burch-Smith, 2015). Stromules (stroma-filled tubules), as the name suggests, are stroma-filled protrusions of both inner and outer plastid envelopes, which emanate from a plastid body (Köhler et al., 1997; Köhler and Hanson, 2000). Shortly after the plastid stroma was first highlighted via fluorescence proteins (Köhler et al., 1997) the formation of stromules was observed under a wide variety of abiotic and biotic stress conditions (summarized in Mathur et al., 2012). Perhaps coincidentally, these are conditions in which communication between the plastid and the nucleus would be essential for maintaining homeostasis.

The most recent and perhaps the most direct evidence for the hypothesized role of stromules in retrograde signaling comes from Caplan et al. (2008, 2015). Not only were stromules induced following expression of the p50 effector (in N-containing *N. bethamiana*), but NRIP1-Cerulean was localized to plastid stroma and observed in stromules which appeared to contact the nucleus. Additionally Caplan et al. (2015) claim that NRIP1 re-targeting from the plastid to the nucleus during HR-PCD (hypersensitive response-programmed cell death) is enhanced in cells when stromules formed connections with the nucleus. This observation has inspired the model that stromules are formed purposefully to amplify/guide retrograde pro-defense signals to the nucleus (Caplan et al., 2015). Assuming that stromules are established to facilitate communication with the nucleus, this raises the question of how the cell supports the establishment of these plastid-nuclear connections. It could be that under certain conditions select plastids sense the nucleus position and intentionally extend a stromule in the direction of the nucleus. A second possibility is that the stromule-nuclear interactions are the result of excessive stromule induction throughout the cell, thus increasing the probability that a subset of stromules would contact the nucleus. If the latter were true, then the functional relevance of these structures in the context of plastid-nucleus interactions would likely be called into question.

In order to understand whether the formation of stromule-nuclear interactions is due to the directional formation of stromules toward the nucleus, or a series of random interactions resulting from very high stromule frequencies, it is essential to analyse the position of all plastids of a given cell, both with and without stromules, relative to the nucleus. A literature survey screening for publications that look into stromule-nucleus relations, revealed that plastids with stromules or stromules themselves have frequently been reported to interact with nuclei (Supplementary Table 1). However despite the many reports of stromule-nucleus interactions, this literature survey revealed that most of these observations were not quantified, and additionally none were considered within the context of the entire cell. This means that although there have been thorough descriptions of plastids and stromules in close proximity to the nucleus the remainder of the cell's plastids/stromules were not considered. The behavior of all plastids and stromules within a cell and their relationship to the nucleus is not well understood, and the question of how such interactions are established by the cell is still open. Evaluating stromule behavior in the whole cell situation is essential for directing speculations about stromule function in the context of nuclear-plastid-stromule communication.

In order to test whether stromules intentionally interact with nuclei or whether those interactions are more likely to be coincidental, whole cell stromule levels, directions and behavior in relation to nuclei were quantitatively evaluated in the upper epidermis of *A. thaliana* rosette leaves. Based on the hypotheses present in the literature that stromules are important to nuclear-plastid communication, the first step was to determine if plastids close to the nucleus are more likely to form stromules than those in more distant regions of the cell, and whether these stromules were facing toward or away from the nucleus. Evidence presented here suggests that there is a 'stromule-promoting zone' surrounding the nucleus, and within this zone it was observed that stromules are largely facing toward the nucleus. Surprisingly, results showed that higher stromule frequencies within this zone are the result of an increase in stromule initiation events, rather than increased stromule stability.

## Materials and methods

### Plant growth conditions

For still images transgenic *Arabidopsis thaliana* stably expressing the transit peptide of ferredoxin-NADPH-oxidoreductase fused to eGFP (*FNRtp:eGFP*) was used. Soil grown plants were exposed to 120 μE m^−2^s^−1^ under short day conditions (8 h light/16 h dark) at 60% relative humidity and 21°C. Leaf discs were collected from the youngest fully expanded rosette leaves of 10–12 week-old plants and placed in a 1.5 mL tube containing water. A vacuum was applied to this tube using a 10 mL syringe placed on top. When the plunger of the tube is pulled water is forced into the intracellular space making microscopy pictures much clearer. This method is also described in Schattat and Klösgen (2011).

For recording time-lapse z-stacks of plastids and nuclei, fully expanded leaves of primary selected 4 week-old *pLSU4::P*_*35S*_*:FNRtp:eGFP:T*_*35S*_*:P*_*UBQ10*_*:H2B:mCherry:T*_*nos*_ (hereafter called *pLSU4::pn*) transgenic plants were utilized. The T_0_ seeds were germinated and incubated for 4 days on AT media (Ruegger et al., 1997) plates containing 40 μg mL^−1^ hygromycin and 250 μg mL^−1^ cefotaxime. Resistant plants were screened for fluorescence and transferred to soil. After 4 weeks of cultivation on soil at 21°C under short day conditions (8 h light/16 h dark) with a light intensity of 120 μE m^−2^s^−1^, mature rosette leaves were harvested and prepared for imaging.

### Acquisition of individual Z-stacks for still images

**Figure 1:**
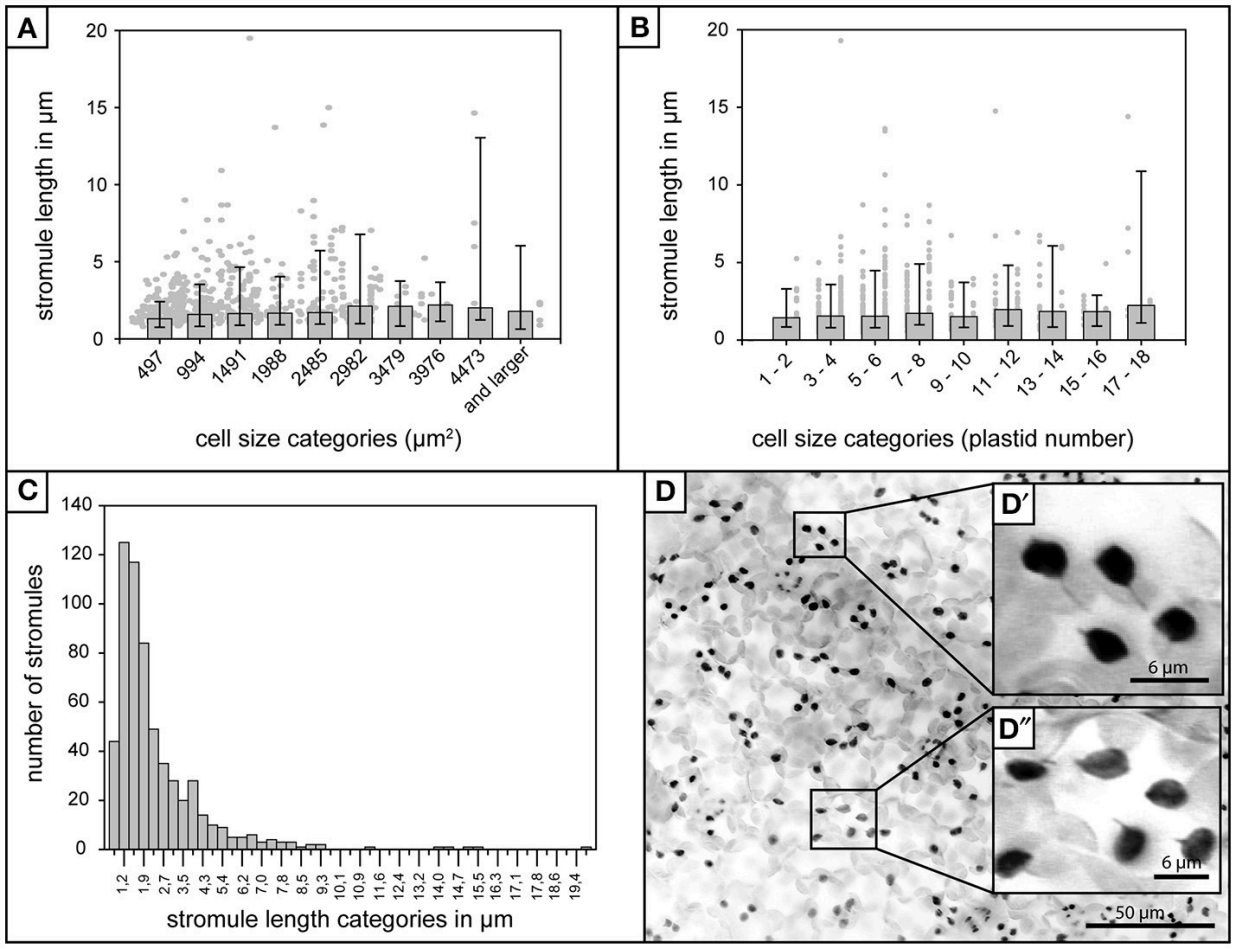
Stromule length in *A. thaliana* rosette leaf upper epidermis. **(A)** Foreground—Bars represent median stromule length for all cell size categories where cell size is expressed in μm^2^ (*n* = 603). Background—Individual stromule length measurements. **(B)** Foreground—Bars represent median stromule length for all cell size categories where cell size is expressed in plastids per cell (same data set as in **A**). Background—Individual stromule length measurements in each category. **(A,B)** Error bars represent 10th and 90th percentiles. **(C)** Histogram of stromule length measurements observed over all cell sizes. **(D)** Z-stack of *A. thaliana* upper epidermis expressing *FNRtp:eGFP* labeling plastids and stromules (converted to grayscale and inverted to increase clarity). Scale = 50 μm. **(D**′**)** and **(D**″**)** insets show examples of several typical short stromules as well as several plastids without stromules (Scale = 6.25 μm).

**Figure 2:**
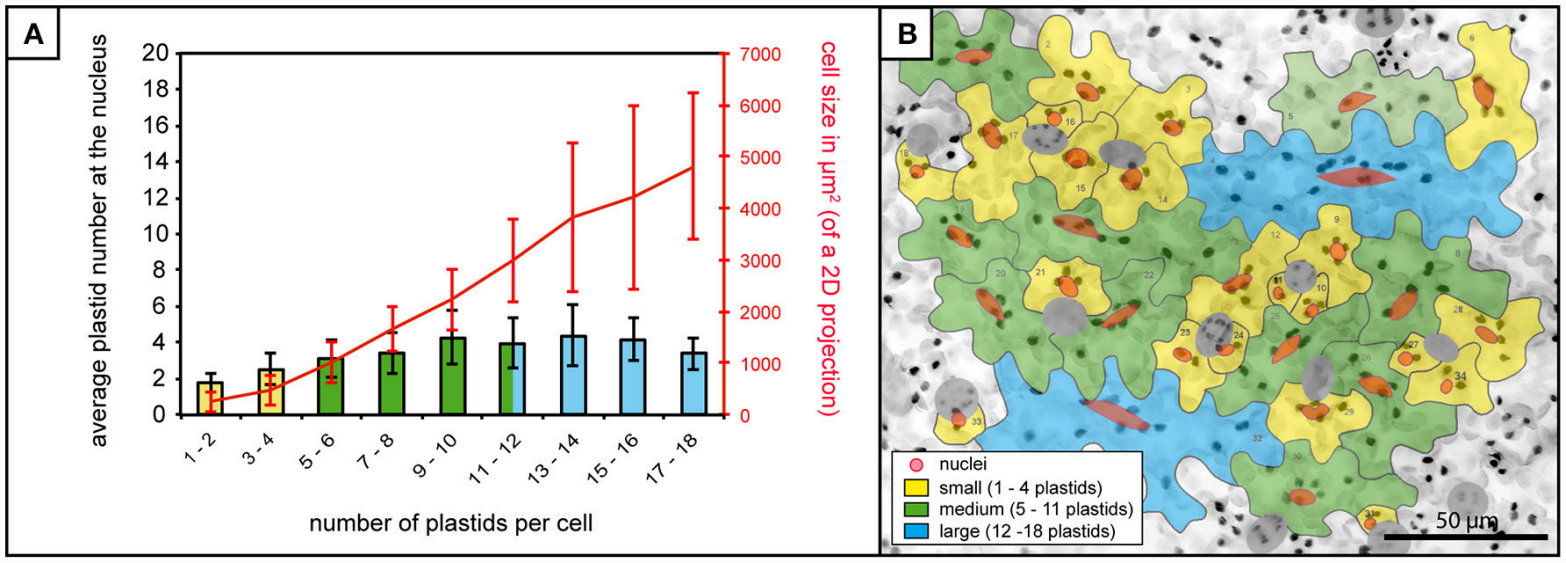
Plastid-nucleus association in *A. thaliana* rosette leaf upper epidermis. **(A)** Bars represent the average number of plastids at the nucleus in cells of increasing plastid number (x-axis), while the red line represents accompanying increases in average cell size. Bars colors match cell size categories, small (yellow), medium (green) and large (blue) cells as shown in **(B)**. Error bars represent standard deviation. **(B)** Z-stack of *A. thaliana* upper epidermis expressing *FNRtp:eGFP* (the same image as in Figure 1D) with cells numbered and outlined in gray, nuclei in red (stomatal guard cells excluded from the analysis, in dark gray). Cells in the three different size categories colored in yellow (small cells), green (medium cells) and blue (large cells). Scale = 50 μm.

**Figure 3:**
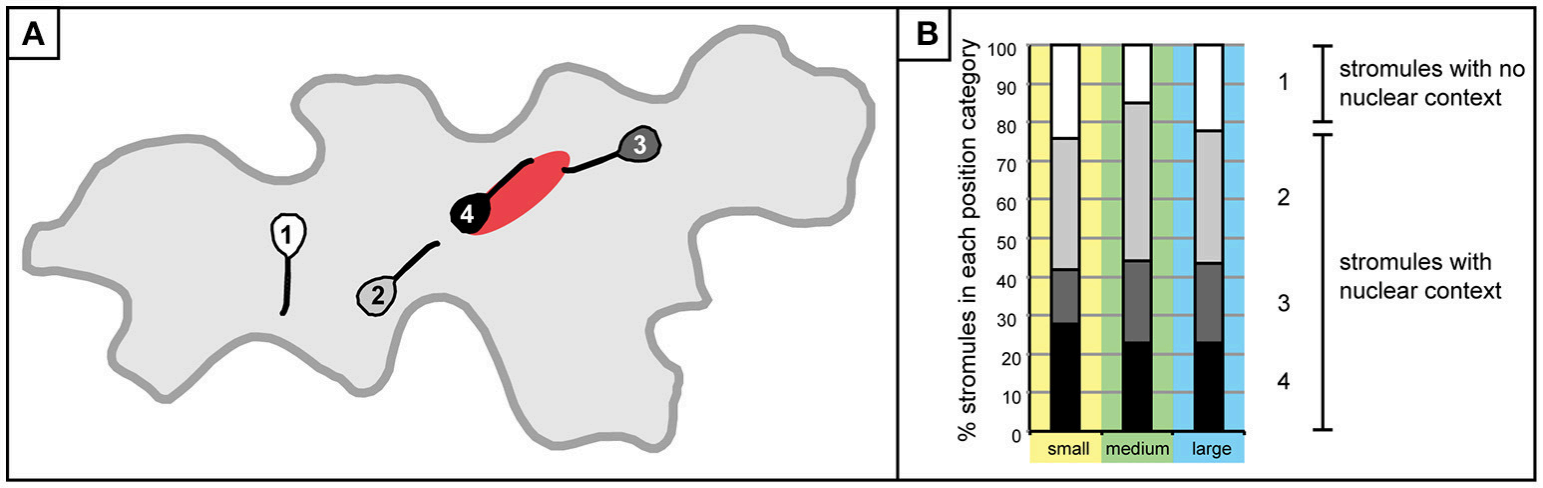
Stromule formation in the context of the nucleus. **(A)** A graphic depiction plastids/stromules within each “position category” created to demonstrate the relationship of a given plastid to the nucleus. Categories were delineated as follows: 1 (white), plastid does not contact the nucleus and stromule does not face or come into contact with the nucleus; 2 (gray), plastid/stromule does not touch the nucleus, but stromule is facing the nucleus; 3 (dark gray), plastid body is not touching the nucleus, but stromule is in contact; 4 (black), plastid body and stromule are in contact with the nucleus. **(B)** Graph depicting the % of plastids harboring stromules within each “position category” in small (yellow; *n* = 432 plastids), medium (green; *n* = 648 plastids) and large (blue; *n* = 154 plastids) cells. Gray scale categories match colors exemplified in **(A)**. Category 1 (white) plastids/stromules show no nuclear context, while the other three categories were classified as “stromules within a nuclear context.”

**Figure 4:**
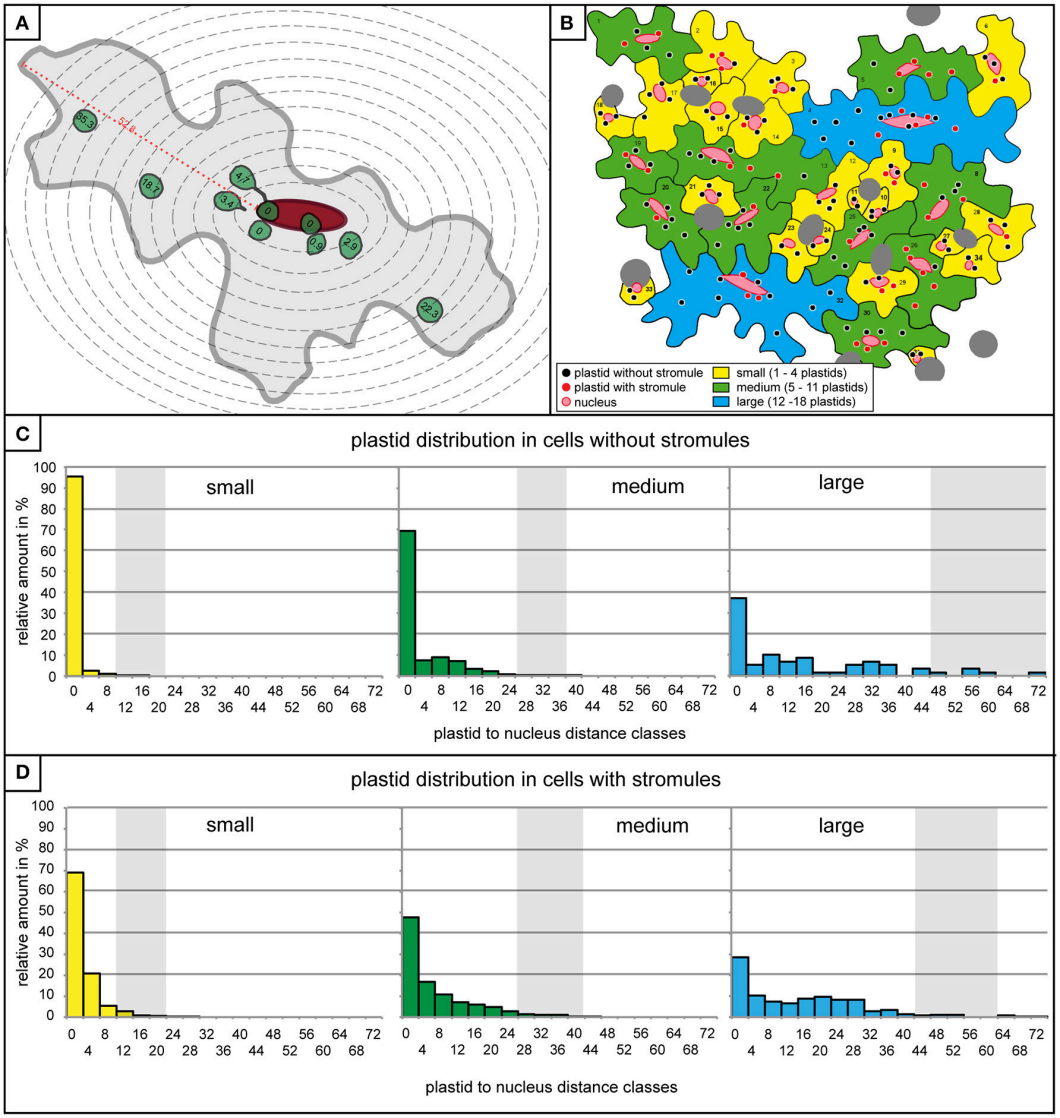
Plastids distribution is similar in cells with and without stromules. **(A)** A graphic representation of a medium sized cell with 4 μm zone boundaries outlined with dotted gray lines, the nucleus in red, and the plastids with and without stromules are in green. Distance of all plastids to the nucleus (in μm) is written within the plastid. The maximum distance possible between a plastid and the nucleus (i.e., the longest distance between the nucleus and the cell border) is highlighted with a red dotted line and marked with the distance measurement for this particular cell. **(B)** A graphic representation of the microscopy picture found in Figures 1D, 2B, demonstrating the distribution of plastids in cells with and without stromules in the different cell size categories. **(C,D)** Histograms exhibiting the number of plastids within each 4 μm zone (x-axis) in small (yellow), medium (green) and large (blue) cells lacking stromules **(C)** and in cells with stromules **(D)**. Thick vertical lines (light gray) represent the average maximum distance a plastid can be from the nucleus. Average maximum distance values were generated by measuring the maximum distance between the nucleus and cell border in each cell and averaging these values (example measurement highlighted by a red dotted line in **A**). The greatest distance possible between the nucleus and the plastid depends on cell size, but also nucleus position within the cell.

**Figure 5:**
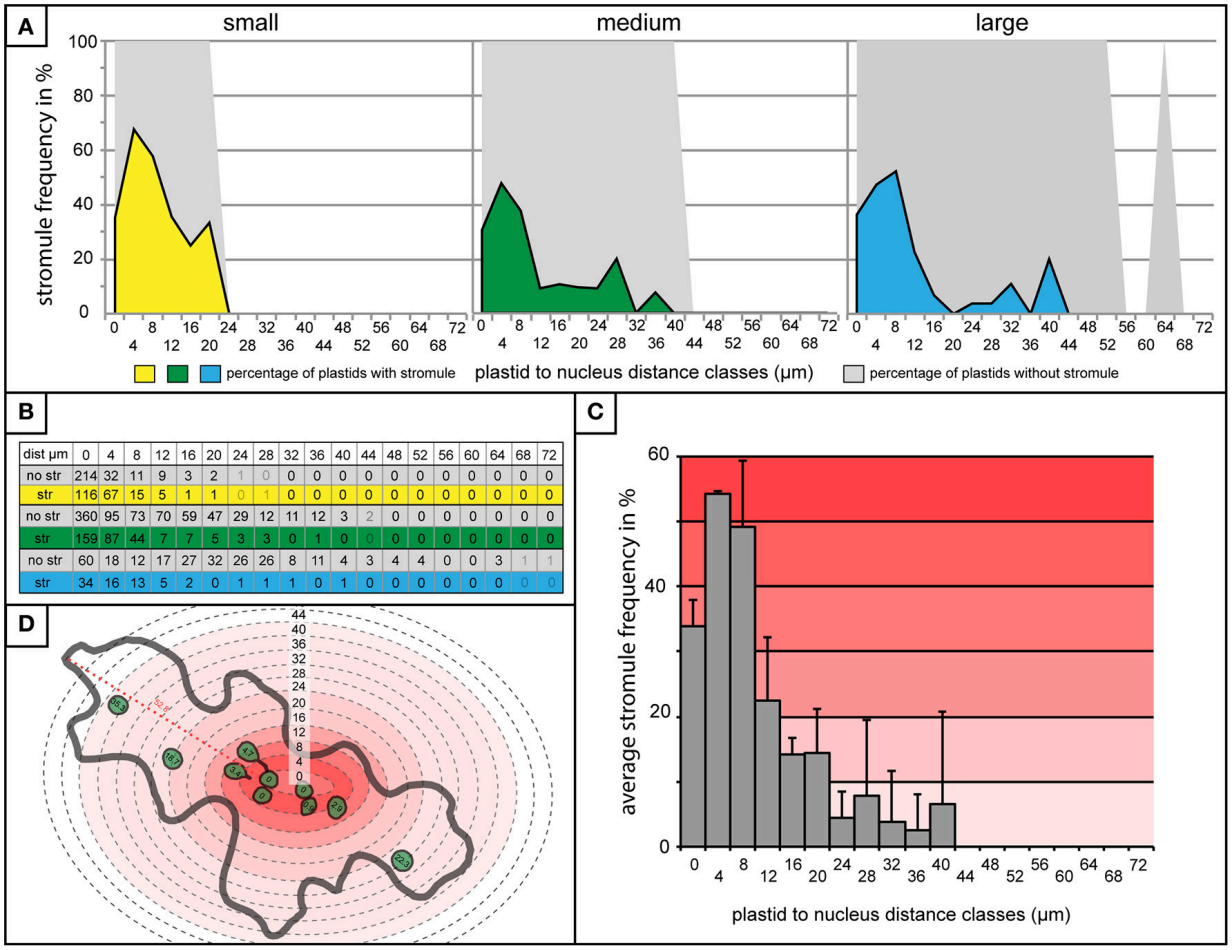
“Stromule-promoting zone” evident within 8 μm of the nucleus. **(A)** Histograms showing stromule frequency in each 4 μm zone at increasing distances from the nucleus (x-axis) in small (yellow), medium (green), and large (blue) cells. Gray areas represent the frequency of plastids without stromules. **(B)** The plastid sample sizes for each 4 μm zone which was illustrated in the histograms in **(A)**. **(C)** Histogram of stromule frequency in each 4 μm zone when data sets from all cell sizes are combined. Highest to lowest stromule frequencies are indicated with a dark-light red gradient background. Error bars represent standard deviation. **(D)** An illustrated example of a cell highlighted according to the stromule frequency gradient outlined in **(C)**. Defined distance classes from histogram in **(C)** superimposed onto illustration (this particular cell has plastids in zones up to 40 μm from the nucleus).

**Figure 6:**
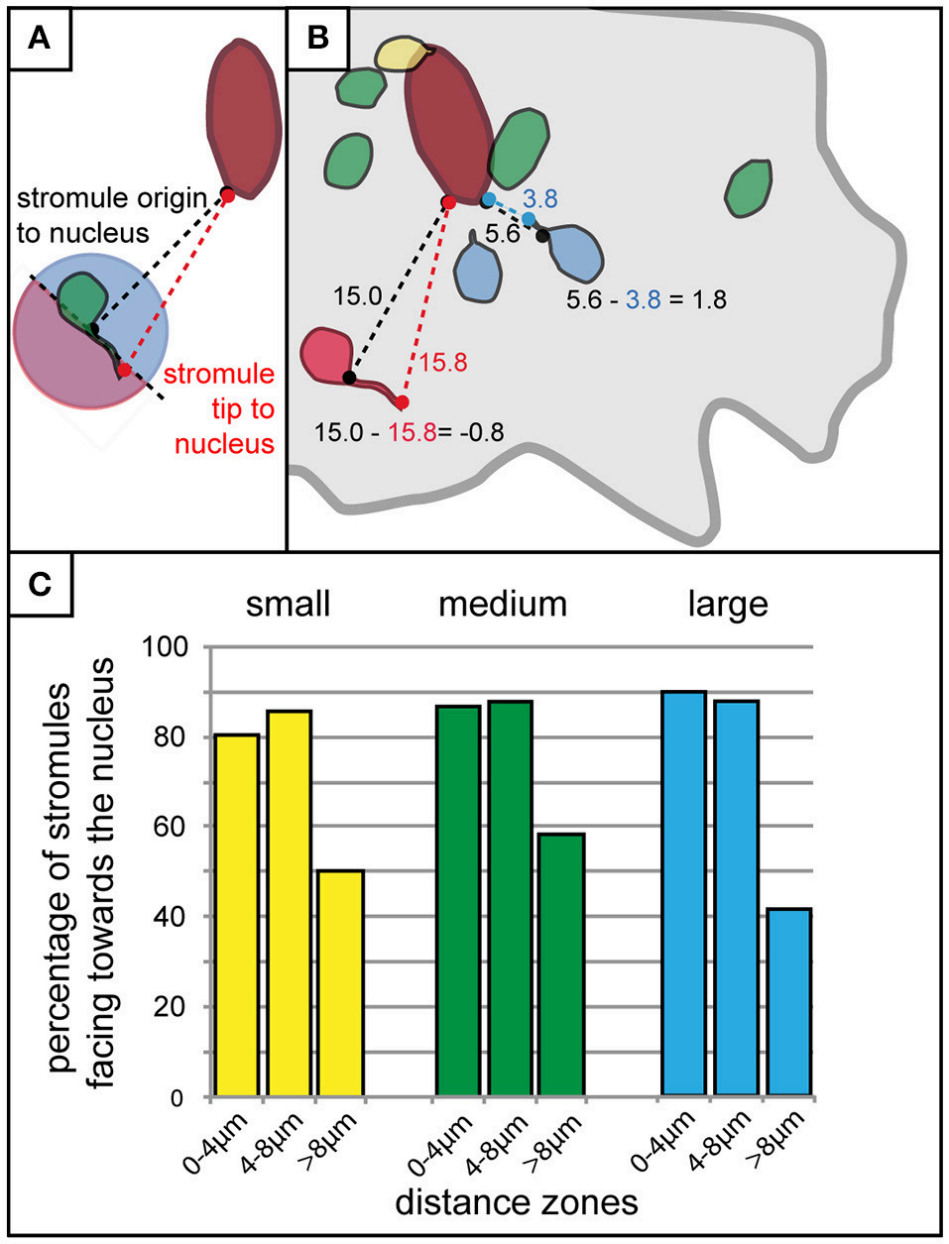
Stromules formed in close proximity to the nucleus usually face it. **(A)** Schematic outline of the method used for the rough determination of whether a stromule faces toward or away from the nucleus. The distance of the base of the stromule as well as the tip of the stromule to the nucleus were measured and compared. If the stromule tip is further from the nucleus than the base of the stromule (any stromule tip falling within the pink part of the circle for the plastid illustrated here) the stromule would be considered facing away from the nucleus, while any tip that is closer to the nucleus than the base (all those falling within the blue part of the circle) would be considered as facing toward the nucleus. **(B)** Illustration of example measurements taken from a real cell showing four plastids without stromules (green), one with a stromule and plastid body touching the nucleus (yellow), two that were considered to be facing the nucleus (blue), and one that was considered to be facing away from the nucleus (red). Example calculations for a stromule facing the nucleus (i.e., stromule decreasing the distance between the nucleus and plastid body), and a stromule facing away from the nucleus (i.e., stromule increasing the distance between the nucleus and the plastid body), are outlined in blue and red, respectively. Cell border outlined in gray, nucleus outlined in dark red. **(C)** Bar chart illustrating the percentage of stromules facing the nucleus (as determined by the criteria outline in **A**,**B**) with increasing distance from the nucleus in small (yellow), medium (green), and large (blue) cells.

Data presented in Figures 1–**6** originates from a re-analysis of a subset of images acquired as part of the work included in Schattat and Klösgen (2011), but the data set presented here is completely original. Images with labeled nuclei, although originally taken along with the images analyzed in Schattat and Klösgen (2011), were not analyzed in that publication. These images document the 4′,6-diamidino-2-phenylindole (DAPI) stained DNA, and effectively label nuclei. These DAPI images were combined with eGFP fluorescence images from Schattat and Klösgen (2011) to analyse stromules/plastid-nucleus relationships.

### Details DAPI staining and imaging

For staining the nuclei in upper epidermis cells, tissue samples were vacuum infiltrated with tap water containing 6 μM (DAPI) and incubated for 10 min at room temperature. A DAPI stock solution was prepared from powder (obtained from Molecular Probes, ThermoFischer Scientific) in double distilled water. Comparison with unstained samples showed that the staining procedure did not change stromule frequency and behavior during these short-term experiments (data not shown). DAPI fluorescence was excited with a HBO100 high-pressure mercury plasma arc-discharge lamp (Carl Zeiss GmbH, Jena, Germany) and imaged using the fluorescence filter set F49 (excitation = G 365; beam splitter = FT 395; emission = BP 445/50) (Carl Zeiss GmbH, Jena, Germany).

### Image processing

Still image stacks were converted into single image projections using CombineZP (Hadley, 2006) and imported into Fiji v.2.0.0-rc-31 (Schindelin et al., 2012). Due to the flat shape of epidermis cells and the location of plastids as well as nuclei on the lower side of cells (facing the mesophyll), 2D projections provide a good representation of the relative position of these organelles in the 3D situation. Measurements of still images were performed via standard analysis tools of Fiji v.2.0.0-rc-31.

### Data analysis

For data handling and analysis Microsoft Excel (version 14.4.4), R (version 3.2.2) and RStudio (version 0.99.484) was used. Statistical tests were performed with SigmaPlot12. Graphs were plotted using Excel or SigmaPlot 12.

### Generation of plastid-nucleus double marker lines

Since the staining of nuclei with DAPI could damage cells during longer experiments and could potentially alter cell bahavior, an alternative fluorescence based strategy was used to label nuclei during long-term movies. To label nuclei Histone-2B (Wozny et al., 2012) was fused to mCherry (Nelson et al., 2007). Expression was driven by the *AtUBQ10* promotor fragment described by Grefen et al. (2010) and terminated by the *NOS* terminator (Nelson et al., 2007). For labeling of the plastid stroma the expression cassette *P*_*35S*_*:omegaleader:FNRtp:eGFP:T*_*35S*_ (described by Marques et al., 2004) was used.

To facilitate efficient cloning of the double protein marker vector Golden Gate cloning was utilized. In order to use this cloning method the *pLSU4* plasmid described by Lee et al. (2012) was turned into a BsaI based, Golden Gate compatible vector. To achieve this the BsaI site existing in the vector backbone was removed by site-directed mutagenesis and the MCS was replaced by two BsaI sites. For cloning the *P*_*35S*_*:omegaleader:FNRtp:eGFP:T*_*35S*_ as well the second expression cassette, *P*_*AtUBQ10*_*:H2B:mCherry:T*_*NOS*_, individual components (e.g., promoters, proteins, tags, and terminators) were amplified via PCR introducing BsaI sites. Following a standard Golden Gate protocol the resultant vector *pLSU4::pn* was produced.

For establishing stable transgenic plants *pLSU4::pn* was transferred into *Agrobacterium tumefaciens* strain GV3101 (pMP90) (Koncz and Schell, 1986) by electroporation. These bacteria were used to transform wild-type *A. thaliana* plants by standard floral dipping (Davis et al., 2009).

### Time-lapse movie acquisition

To prevent leaves from drying out during movies a chamber was created by applying a c-shaped string of silicon grease to a glass slide. The leaf section was placed with a drop of tap water in the middle of the silicon “c” and covered with a glass coverslip (High Precision No. 1.5H 170 ± 5 μm). Excess water was removed from under the coverslip using a paper towl, thus allowing for gas exchange.

Imaging was done using a Zeiss LSM 780 microscope (Carl Zeiss GmbH, Jena, Germany) based on an inverted AxioObserver and equipped with a 63× lens (C-Apochromat 63×/1.20 W Korr M27, Carl Zeiss GmbH, Jena, Germany). Fluorescence was induced via a 488 nm laser line of a multiline argon laser (25 mW) and a 561 nm laser. Fluorescence was recorded in single-track mode. Individual channels for eGFP (493–556 nm), for mCherry (596–623 nm) and for chlorophyll (653–758 nm) were defined. The transmitted light image was recorded in a separate channel. In preparatory work laser intensity (488 nm 1.8%; 561 nm 2%), pixel dwell time (0.79 μs), line averaging (2 times), pixel number (1,024 × 1,024) as well as the frame rate (3 min) was optimized to minimize the impact of laser light on the cells. Based on its sensitivity to bleaching, the stability of the chlorophyll fluorescence was used to estimate the impact of the laser light on the plant tissue. The microscope setup was controlled with the microscope manufacturer's software Zen (Carl Zeiss GmbH, Jena, Germany).

### Time-lapse movie processing

A total of 7 movies were acquired. Data clearly showed that cell size does not have an impact on the stromule-promoting zone, so it was decided that the analysis would be focused only on medium and large cells. Movies recorded the behavior of 252 plastids plastids in 22 cells. The average plastid number per cell was 11.45 (standard deviation = 5.79; maximum = 30 plastids; minimum = 5 plastids). For analysis, 4D imaging data (x/y/z/t) was projected in 3D (x/y/t) by creating a maximum intensity projection along the z-axis for each time point using Fiji v.2.0.0-rc-31 (Schindelin et al., 2012).

### Time-lapse movie data analysis

Movie analysis began by outlining all whole cells of interest and labeling them with an ID number. Subsequently the position of each plastid was tracked in relation to the nucleus across 30 frames. For plastids the coordinates of the point closest to the nucleus was tracked, and for nuclei the point closest to the respective plastid was tracked. When plastid and nucleus fluorescence overlapped it was assumed that both organelles “touch” and only one set of coordinates was recorded. The “ManualTrack” PlugIn of Fiji v.2.0.0-rc-31 (Schindelin et al., 2012) was utilized for point tracking. The x-y coordinates provided by the “ManualTrack” PlugIn were used to calculate the Euclidean distance between both points in pixels and subsequently converted to μm.

## Results

### *A. thaliana* upper epidermis as a model system

Before beginning the analysis of stromule-nuclear interactions in the whole cell context an appropriate model tissue had to be chosen, taking into account any tissue specific influences on stromule frequency. Hypocotyl epidermal cells are very frequently used in the study of stromules. However, Waters et al. (2004) observed that in dark grown tobacco hypocotyl tissue increases in cell size are correlated with a decrease in plastid density, and that this decrease in plastid density is correlated with increases in stromule abundance. Taking this finding into account, it had to be ensured that the parameters relating to plastid and stromule position would not be heavily influenced by cell size in the model tissue. *A. thaliana* rosette leaf upper epidermis does not exhibit such a correlation, and so was chosen as the model tissue for the following experiments. Schattat and Klösgen (2011) demonstrated that although upper epidermis cells are hugely variable in size, it is clear that the plastid density is constant and that stromule frequency is unlikely to be correlated with cell size. Regardless of cell size, stromule frequency (number of plastids with stromules ÷ total plastids) of the upper epidermis largely ranges between 0.13 and 0.23 (Schattat and Klösgen, 2011). Plastids are not saturated with stromules, providing the chance to examine the behavior and position of plastids both with and without stromules. Both the independent nature of stromule frequency and cell size, as well as moderate stromule numbers make *A. thaliana* upper epidermis ideal for this study. It should be noted that microscopy images collected by Schattat and Klösgen (2011) were well suited for this analysis, and were re-evaluated for the purposes of this publication. These experiments utilized 10–12 week-old transgenic *A. thaliana* (Columbia ecotype) rosette leaves stably expressing an *FNRtp:eGFP* plastid label (*FNRtp:eGFP* 7–4; Marques et al., 2003, 2004) and nuclei were labeled with DAPI.

### Most stromules in the upper epidermis are short

In addition to the influence of cell size and plastid density on stromule number, Waters et al. (2004) also found that stromule length was influenced by these factors, with larger cells/lower plastid density resulting in longer stromule lengths in tobacco hypocotyl. Although the upper rosette leaf epidermis has a constant plastid density the relationship between stromule length and cell size also needed to be assessed to ensure the independence of these two variables. The length of all stromules present in all the images collected (*n* = 603) were measured and it was found that, in contrast to tobacco hypocotyl, *A. thaliana* leaf upper epidermal tissue showed no obvious correlation between median stromule length and the cell size or number of plastids per cell (Figures 1A,B). In fact, median stromule lengths indicate an abundance of very short stromules (between 1 and 2 μm) in all cells (Figure 1C). Interestingly, the longest stromule observed (close to 20 μm) was not in the largest cell size class, but was in a cell with only 4 plastids (Figure 1B). A histogram of stromule length measurements encompassing all cell sizes shows a drastic right skew, a median value of 1.9 and an arithmetic mean of 2.7 (Figure 1C), thus confirming that in *A. thaliana* upper epidermis, stromules are most often short. It should be noted that stromule measurements in this tissue were simplified by the fact that stromules showed no branching (Figure 1D), a phenomenon that is described in other tissues, such as *A. thaliana* cortical root tip and *N. benthamiana* hypocotyl epidermis and leaf epidermis (Schattat et al., 2011).

### The relationship between plastids and nuclei in the upper epidermis

In order to assess the potential for a relationship between the plastids/stromules and the nucleus of cells the first step was to quantify where the plastids are normally positioned in the cell. In this way it could be determined whether plastids (with or without stromules) preferentially congregate around the nucleus, or whether they are equally distributed throughout the cell. The total number of plastids per cell was counted as well as the number of plastids that appeared to be “in contact” with the nucleus. Based on the analysis of 1,213 cells in 80 images from 8 plants it was found that 99% of cells have at least one plastid in contact with the nucleus. Although plastids at the nucleus increased slightly as plastid number/cell size increased (Figure 2A) the mean number of plastids at the nucleus remained below 5 in all cases, despite the fact that larger cells could harbor up to 18 plastids (Figures 2A,B). This shows that there is consistently a subpopulation of plastids at the nucleus, which increases little with increases in cell size/plastid number.

### Stromule orientation gives the impression that stromules “reach” for the nucleus

Initial efforts were made to establish a clearer picture of how plastids are distributed, and revealed that although there are usually a few plastids at the nucleus, there are also plastids scattered throughout the cell. To evaluate the feasibility of the hypothesis that stromules are formed for the purpose of contacting the nucleus or the cell periphery the next step was to examine how stromules emanating from these plastids were positioned relative to the nucleus. For this analysis only cells with stromules could be assessed, resulting in a decrease in the size of the data set. Of the 1,213 cells that were analyzed for the first experiments 567 cells had one or more plastids with stromules. These cells were divided into three size categories so as not to exclude the possibility that stromule orientation could be influenced by the size of the cell. Small cells were defined as those that had a maximum of 4 plastids, medium cells harbored 5–11 plastids, and large cells harbored 12–18 plastids. Size categories were chosen in a way so as to ensure an adequate number of cells were in each category (small cells, *n* = 700; medium cells, *n* = 469; large cells, *n* = 44). In order to evaluate the relative position and orientation of stromules in regard to the nucleus each plastid with a stromule was visually examined and sorted into one of four categories: (1) plastids and stromules which show no nuclear context, where the plastid body is not in contact with the nucleus and the stromule does not face the nucleus, (2) the plastids and stromules are not in contact with the nucleus, but the stromule is facing toward the nucleus, (3) the plastid is not in contact with the nucleus, but the stromule is “connecting” the plastid to the nucleus, and 4) the plastid and the whole stromule are in contact with the nucleus (Figure 3A). Analysis of stromule position relative to the nucleus showed a similar trend in all cell size classes. Most stromules (75–85%) can be found in a nuclear context, meaning that in most instances stromules are either “reaching toward,” or touching the nucleus, while the remaining stromules show no affinity for the nucleus (Figure 3B).

### Identification of a “stromule-promoting zone”

The observation that stromules often form within the context of the nucleus, either touching it or “reaching” toward it, provides support for the idea that stromules act to minimize the distance between these two organelles. Given that in this model tissue most stromules are short, it was hypothesized that there is a relatively small distance within which a stromule would be useful for the purpose of communicating with the nucleus. If this were the case, then it would be expected that plastids closer to the nucleus are more likely to form a stromule than those in more distant regions of the cell, given that for these distant plastids the nucleus is effectively “out of reach.” To assess whether this is the case, the entire data set was re-examined and the distance between all plastids and the nucleus was measured in cells both with and without stromules. Due to variations in image quality not all cells could be included in this very detailed analysis reducing the number of cells considered to *n* = 527 cells with no stromules (*n* = 2,132 plastids) and *n* = 325 cells harboring stromules (*n* = 1,898 plastids). Since stromule frequency can only be calculated for a population of plastids and not for an individual plastid they had to be grouped into populations based on their distance from the nucleus. For this purpose zones of increasing distance around the nucleus were defined, each zone being 4 μm (example cell shown in Figure 4A), which corresponds to the average size of a pavement cell plastid (average plastid size = 3.8 μm, *n* = 4,834, standard deviation = 0.8). Plastids touching the nucleus were placed into their own distance class. Figure 4B shows an animated example of the distribution of plastids with and without stromules in an average microscopy image.

Although it had already been determined that most cells are likely to have at least one plastid near the nucleus independent of whether they had stromules or not (Figure 2A), quantification of plastid position using the abovementioned zones provided more precise idea of what these two cell types looked like. Prior to evaluating whether plastids near the nucleus were more likely to show higher stromule frequency, cells with and without stromules were evaluated to determine whether they exhibit comparable plastid distributions. Again, data was divided according to the three cell size categories, small, medium and large (a graphic representation of a microscopy picture outlining the three types of cells can be seen in Figure 4B). The result was that cells both with and without stromules showed a similar plastid distribution (Figures 4C,D). Based on distance measurements, plastids can be found at almost every possible distance to the nucleus in cells with and without stromules. However, the large majority of plastids are categorized as touching the nucleus (cells with no stromules: small cells = 95.5%, medium cells = 69.4%, large cells = 37.3% and in cells with stromules: small cells = 69%, medium cells = 47.7%, large cells = 28.4%), or can be found within 8 μm of the nucleus (no stromules: small = 99.4%, medium = 85.5%, large = 52.5%, with stromules: small = 95.2%, medium = 75.1%, and large = 46.2%). In both cell types and in all cell sizes there was a sharp decline in the number of plastids in zones at greater distances from the nucleus. One difference between these two cell populations is that there is a noticeably higher frequency of plastids touching the nucleus in cells without stromules (compare Figures 4C,D). Overall, the quantification of plastid position in cells with and without stromules revealed that zones further from the nucleus harbor fewer plastids in both cell types. These results suggest that plastid position does not have to change drastically to facilitate stromule induction.

Finding very little difference in plastid distribution between cells with and without stromules, it was then possible to use data from cells with stromules to evaluate whether stromule frequency is higher in zones closer to the nucleus. To do this, stromule frequency was calculated for each zone of 4 μm for small, medium and large cells (Figure 5A). Zones with less than three plastids present were eliminated based on an insufficient sample size (number of plastids with an without stromules in each zone outlined in Figure 5B). The result of this analysis, in the case of all three cell size categories, was that plastids close to the nucleus were up to 10 times more likely to form stromules than those in more distant zones (Figure 5A). Peak stromule frequencies of 68, 48, and 52% (for small, medium and large cells, respectively) were observed to fall within 8 μm of the nucleus and dropped drastically thereafter (Figure 5A). When data from all three cell sizes are combined the same trend is seen (Figure 5C). These results demonstrate that stromule frequencies clearly differ between zones, with most stromules specifically occuring in zones close to the nucleus. Independent of cell size, stromule formation is favored within ~8 μm of the nucleus and as a result this region was appropriately deemed the “stromule-promoting zone.”

### Plastid position relative to the nucleus influences stromule orientation

It was clear that the majority of stromules were found in the context of some kind of nuclear interaction, and that stromules were more likely to form within 8 μm of the nucleus. What was not clear was whether the distance of a plastid/stromule from the nucleus influences the direction the stromule is facing. The next step was to roughly determine whether stromules formed in close proximity to the nucleus were more likely to face it. To achieve this each plastid with a stromule was evaluated via measuring the distance between the nucleus and the stromule origin, as well as the distance between the nucleus and the stromule tip (Figure 6A). If the difference between these two values (nucleus to stromule origin—nucleus to stromule tip) was greater than 0 the stromule was said to point toward the nucleus (decreasing the distance between the plastid and the nucleus), while values less than 0 led to the conclusion that the stromule pointed away from the nucleus (Figure 6B). Results for all cell sizes showed that within the stromule promoting zone 80–90% of all stromules decrease the distance between the plastid and the nucleus (Figure 6C). In zones at greater distances from the nucleus stromule direction appeared to become random (50% of stromules faced the nucleus), or they became more likely to face away from the nucleus (<50% of stromules faced the nucleus). It can be concluded that the frequency of stromules, and the orientation of stromules are influenced by the position of the plastid relative to the nucleus.

### Increased stromule frequencies near the nucleus are the result of increased stromule initiation events, not an increase in stromule stability

Through the detailed characterization of stromule formation within the whole cell context via still images it has been established that stromules preferentially form around the nucleus. This provides the first quantitative support for the hypothesis that interactions between stromules and nuclei are not solely coincidental but could be functionally relevant, thus confirming the suspicions of many scientists (refer to Supplementary Table 1). Assessment of still images clearly suggested that stromules “reach” for the nucleus, with the potential for retrograde signal transfer. However, this raised a host of mechanistic questions regarding how stromule enrichment is achieved within this 8 μm zone and how stromules are so efficiently directed as they extend toward the nucleus.

**Figure 7:**
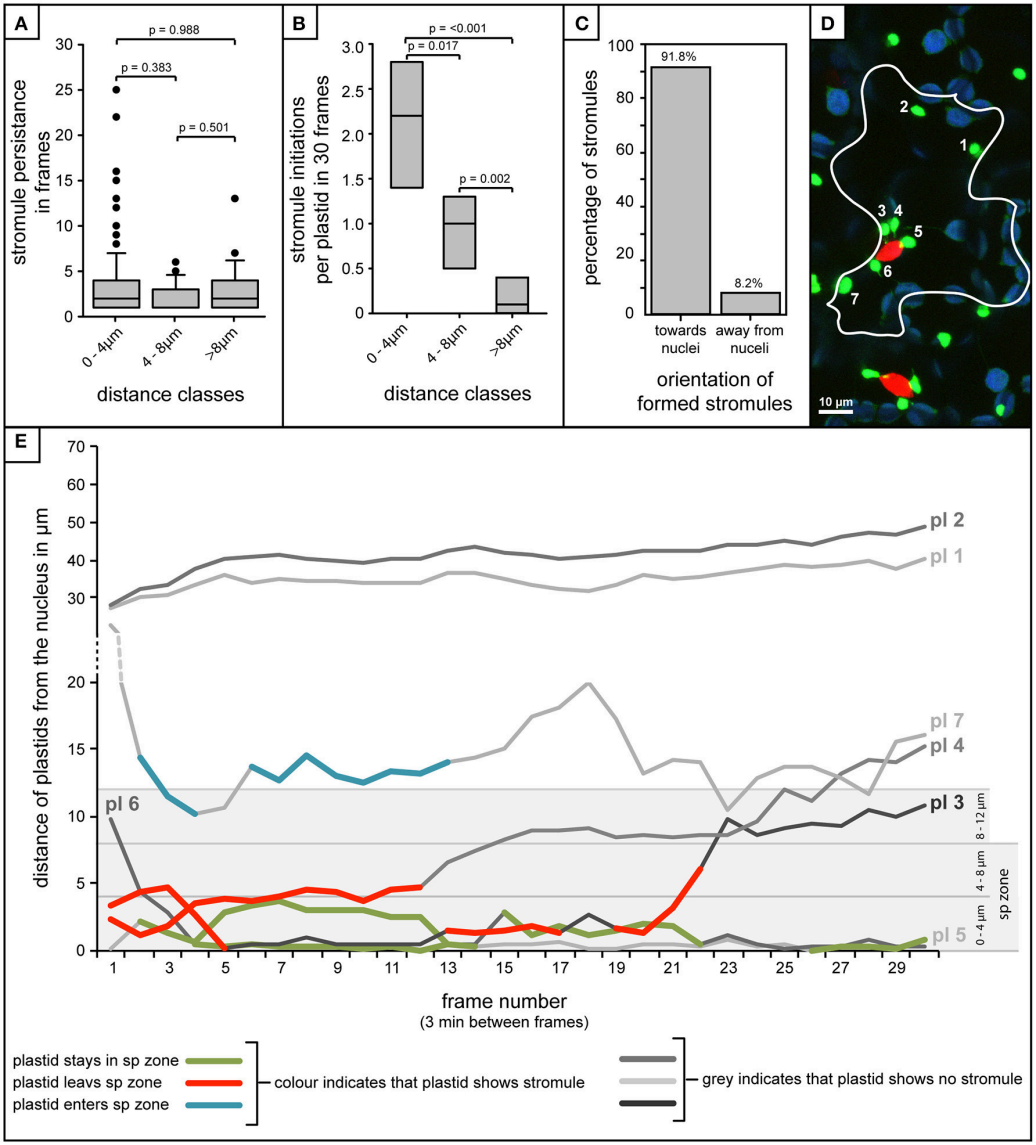
Stromule enrichment in close proximity to the nucleus is due to an increase in formation events, and not to an increase in stromule persistence over time. **(A–C,E)** Quantification of stromule behavior resulting from the frame-by-frame analysis of 90 min movies (30 frames, 3 min/frame). **(A)** Box plot showing the persistence of stromules with increasing distance from the nucleus as measure by the number of frames individual stromules persist for. Boxes represent 50% of all data, horizontal line within boxes represents the median, and error bars represent 90th percentile. *p*-values resulting from Rank-Sum tests comparing treatments can be found above brackets identifying treatments used for comparisons. **(B)** Box plot representing the number of stromule initiations observed per plastid in each distance class over a period of 90 min (*n* = 7 videos). Boxes represent 50% of all data, median is represented by a horizontal line within boxes. *p*-values resulting from Rank-Sum tests comparing treatments can be found above brackets identifying treatments used for comparison. **(C)** Percentage of stromules facing toward and away from the nucleus (method for orientation evaluation outlined in Figures 6A,B). **(D)** Example of a single frame from a movie with a single cell outlined. Bright green plastids and red nuclei are organelles of the upper epidermis of an *A. thaliana* wild-type plant transformed with the *pLSU4::pn* plasmid (blue channel, chlorophyll fluorescence; red channel, *H2B:mCherry* fluorescence; green channel, *FNRtp:eGFP* fluorescence). Plastids were assigned numbers to track their identity over the course of the movie (also seen in white). Larger, round, blue signals represent underlying mesophyll plastids, where chlorophyll signal is brighter than the *FNRtp:eGFP* fluorescence signal. **(E)** Graph representing the movement of each plastid relative to the nucleus in the cell shown in **(D)** over the course of 30 frames/90min (Supplementary Movie 1). Each line represents a different plastid (labeled “pl #”), assigned numbers are shown in **(D)**. Gray scale lines represent frames where the plastid has no stromule, while colored lines represent frames in which stromules are observed. The line with blue segments (pl 8) represents a case where the plastid starts out away from the nucleus, and as it moves closer stromules are initiated, while red lines (pl 3 and pl 4) both illustrate plastids that start close to the nucleus with a stromule, and following movement away from the nucleus they lose their stromule. Green indicates plastids that stay within the “stromule-promoting zone,” initiating one or more stromules over the course of the movie (pl 5 and pl 6).

The analysis of over 80 individual z-stacks has provided us with snapshots of stromule behavior in time. Snapshots, although very useful for evaluating stromule frequency, do not give us any information about the mode with which this enrichment is occurring. It was therefore postulated that enrichment of stromules in the stromule-promoting zone could be the result of two different scenarios; an increase in stromule stability or more frequent stromule initiation events. To assess the feasibility of these hypotheses required the collection of long-term movies via a confocal microscope, using a new *A. thaliana* transgenic line highlighting both plastids (*FNRtp:eGFP*) and nuclei (*H2B:mCherry*). To determine whether stromules are more stable near the nucleus the persistence of stromules at different distances from the nucleus was measured over 90 min (plastids *n* = 252, cells *n* = 22, videos *n* = 7). Surprisingly, movies revealed that stromules were most often present for between 1 and 4 frames (30 frames, 3 min between frames) independent of how far they were from the nucleus (Figure 7A). This analysis suggests that higher stromule frequencies near the nucleus are not the result of increased stromule stability. The same videos were re-evaluated to determine whether there was a difference in the number of stromules initiated in the different zones. The average number of stromules formed per chloroplast was measured over 90 min. It was revealed that plastids closest to the nucleus showed the highest number of stromule initiation events, with a steady decrease in initiations with increasing distance from the nucleus (Figure 7B). At the same time the direction of newly initiated stromules was recorded, revealing that within the stromule-promoting zone over 90% of newly formed stromules face toward the nucleus (Figure 7C). These results suggest that the reason more stromules are observed close to the nucleus in snapshots is the direct result of an increase in stromule initiation events, and not due to increased stromule persistence.

### Movement in and out of the zone is correlated with the ability of plastids to form stromules

A few plastids observed were capable of moving in and out of the stromule-promoting zone and lost or gained a stromule accordingly. For example, occasionally plastids started out in the stromule-promoting zone with a stromule, sometimes exhibiting several stromule initiations and retractions, and then moved away from the nucleus, immediately losing their stromule, and failing to form another (Figures 7D,E pl3 and pl4; in Figure 7E the red marked lines). Additionally there were cases where more distant plastids started with no stromule, but gained a stromule when they entered the zone (example in Figure 7E, pl7 highlighted in blue). Movement into and out of the stromule-promoting zone was not always a result of the plastid changing its position, but also occurred when the nucleus changed position, moving either closer or further from plastids.

## Discussion

It has been suggested that so-called “retrograde” signals are sent from the plastid to the nucleus to deliver important information regarding the physiological status of these endosymbiotic organelles. In recent years a number of potential retrograde signals have been identified, several of which have been suggested to travel to the nucleus via stromules (Caplan et al., 2015), thus implying the intentional extension of stromules for the purpose of communicating with the nucleus. However, based on current data available in the literature it is unclear whether interactions between stromules and nuclei are intentional or observed as a result of an overall increase in stromules throughout the cell, thus increasing the probability that stromules contact the nucleus.

To evaluate whether nucleus-stromule contacts were coincidental, rosette leaves of *A. thaliana* upper epidermis were chosen as a model. Thorough characterization of this tissue has led us to conclude that observed interactions between stromules and nuclei are not coincidental. In fact, a definitive “stromule-promoting zone” was discovered within 8 μm of the nucleus. Within this zone almost all stromules are either facing and/or touching the nucleus (80–90%), while plastids outside this zone are both less likely to form stromules, and stromules that do form appear to lack directionality. Increased stromule frequency in the stromule-promoting zone was further determined to be the result of an increase in stromule initiation events as opposed to increased stromule stability in close proximity to the nucleus. These observations raise two questions, the first being how the stromule-promoting zone and stromule directionality is established and maintained, and the second being whether these interactions are truly relevant in the context of retrograde signaling.

### The mechanism behind the stromule orientation within the stromule promoting zone

**Figure 8:**
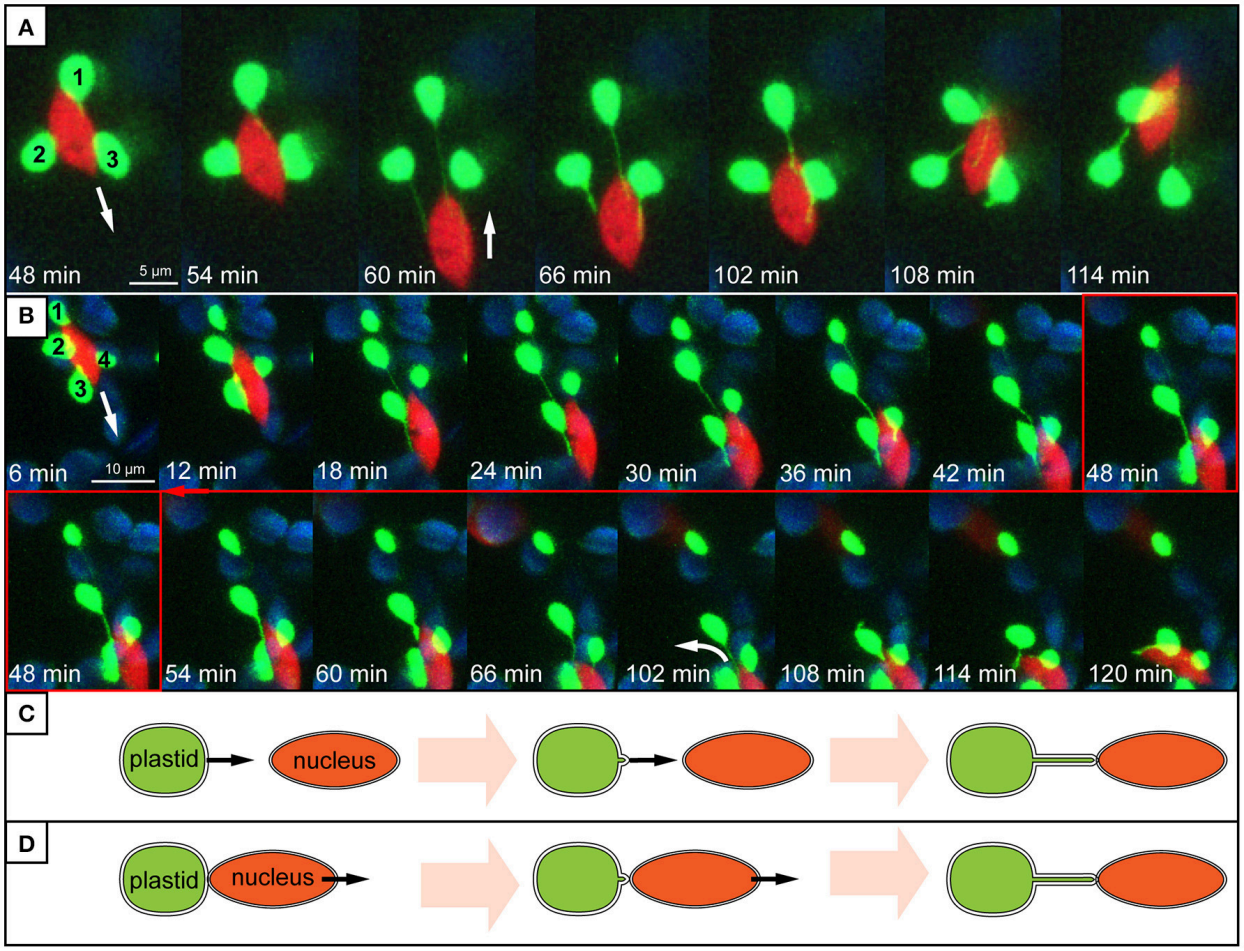
Stromule behavior seems linked to nucleus movement. **(A,B)** Images are maximum intensity projections along the z-axis, taken from a time series of z-stacks obtained by a confocal laser-scanning microscope. Bright green plastids and red nuclei are organelles of the upper epidermis of an *A. thaliana* wild-type plant transformed with the *pLSU4::pn* plasmid (blue channel, chlorophyll fluorescence; red channel, *H2B:mCherry* fluorescence; green channel, *FNRtp:eGFP* fluorescence). The white arrows indicate the direction of nuclear movement. Larger, round, blue signals represent underlying mesophyll plastids, where chlorophyll signal is brighter than the *FNRtp:eGFP* fluorescence signal. **(A)** Series of images taken from Supplementary Movie 2. It demonstrates how nucleus movement “pulls” stromules out of a group of stationary plastids (1–3) and how stromules shorten, when the nucleus changes its direction. **(B)** Series of images from Supplementary Movie 3 showing how a nucleus moves away from a group of plastids (1–4). Plastids 2–4 keep their extended stromules and follow the moving nucleus. Plastid 1 stays stationary and looses its stromule after the nucleus moved more than 12 μm away from it. **(C,D)** Schematic representation of two possible ways how nucleus associated stromules might be formed. **(C)** Scenario 1: stromules are formed by plastids residing further away from the nucleus growing toward (“reaching out”) and connecting to the nucleus. **(D)** Scenario 2: nucleus associated plastids are anchored to the surface of the nucleus and due to nucleus movement a stromule is pulled out of the surface of the stationary plastid.

Evidence presented here suggests that stromule behavior/formation is heavily influenced by the proximity of plastids to the nucleus. Within the stromule-promoting zone the majority of stromules observed were found to face in the direction of, or directly contact the nucleus. The directionality of stromules toward the nucleus that was observed in still images could be intuitively interpreted as an attempt by plastids that do not contact the nucleus, to “reach out” for the nucleus in order to establish direct contacts. This would imply that plastids “sense” or “know” where to find the nucleus. However, none of the stromules observed in movies demonstrated such clear directional growth toward the nucleus. Instead stromule formation seemed to be correlated with plastid and nucleus movement. Frequently plastids appeared to be anchored with their membrane near or at the nucleus, with the extension of a stromule occurring when there was an increase in distance between the organelles. Movement of the nucleus away from the plastid body often resulted in the formation of stromules that appeared to be “reaching” for the nucleus (Figure 8A, Supplementary Movies 2, 3). In movies it is clear that stromule tips are anchored at a point near or at the nucleus, so that wherever the nucleus moves the directionality of stromules (toward the nucleus) is maintained. Given that 90% of stromules within the stromule-promoting zone are formed facing the nucleus, this provides a convincing mechanistic explanation for most of the stromule formation observed. Although preliminary, this data implies that non-perinuclear plastids within the stromule-promoting zone are not likely forming stromules toward the nucleus to transmit a signal, but rather as a result of the independent movement of both organelles.

### Actin distribution and rearrangement as an explanation for the stromule-promoting zone

Currently there is a consensus in the literature that cytoplasmic cytoskeletal elements are essential for maintaining wild-type stromule levels and morphologies. Although the involvement of microtubules in stromule formation appears to differ among tissues/plastid types (Kwok and Hanson, 2003 vs. Holzinger et al., 2007; Natesan et al., 2009), it seems that the involvement of actin is more universally accepted. Actin filaments have been shown to be essential for stromule maintenance in multiple tissues, such as etiolated *Nicotiana tabacum* hypocotyl epidermis (Kwok and Hanson, 2003), *Oxyria digyna* mesophyll (Holzinger et al., 2007), peels of tobacco leaf lower epidermis and onion bulb epidermal cells (Natesan et al., 2009). Kwok and Hanson (2003) found that following treatment with Cytochalasin D (microfilament inhibitor) and Latrunculin B (actin polymerization inhibitor) stromule frequency was reduced, and those that did remain were shorter and thicker than in untreated tissue. Some of the stromules remaining following CD and LatB treatment formed loops (Kwok and Hanson, 2003), as if they had been anchored, extended/stretched and then released from actin to form a loop of slack membrane at the plastid surface, a formation also described in *Iris unguicularis* petals as a “remnant loop” by Gunning (2005). Observations made following actin inhibitor treatments cumulatively suggest that stromules are anchored to actin, and this acts to maintain stromule length, width, number and movement.

Unequal distribution of actin throughout the cell could offer one explanation for why stromules are preferentially enriched when plastids are close to the nucleus. If actin is key to the elongation of stromules, then this could mean that more actin around the nucleus leads to a higher abundance of potential stromule anchor or initiation sites, and so increases the probability that the plastid would initiate a stromule. In support of this hypothesis *A. thaliana* mesophyll cells show high actin densities in close proximity to the nucleus (Kandasamy and Meagher, 1999). Cryofixatation of leaves followed by immunofluorescence localization of actin reveals that a high-density basket of thin actin filaments exists around the nucleus (Kandasamy and Meagher, 1999). In pavement cells as well as mesophyll cells the nucleus is also associated with thicker actin bundles that run along the length of the cell (Kandasamy and Meagher, 1999; Higa et al., 2014). From these bundles a series of thin actin filaments extend in random directions into the cell cortex (Kandasamy and Meagher, 1999). Assuming that the likelihood of forming a stromule is positively correlated with the number of actin contact sites, the decreased actin density in this region could, at least in part, cause a decrease in stromule appearance in the cell cortex.

Based on time-lapse movie data, the abundance of stromules near the nucleus appears to be promoted by both a highly mobile nucleus and, perhaps, high actin density in this region. In other words there are many potential actin-anchoring sites available for plastids within the stromule-promoting zone, and the movement of both the nucleus and the surrounding actin is generating the “force” necessary to form stromules. Live-cell imaging of pavement cells has shown that the actin basket surrounding the nucleus is constantly moving together with the encaged nucleus and closely associated plastids (Higa et al., 2014). Movies confirm this rigorous nucleus movement. Based on movie data it could be hypothesized that in cases where plastids do not immediately follow the moving nuclei, plastid-actin contact sites are moved away from the plastid body and thereby “pull” out stromules. Future work will need to utilize actin labeled stable transgenic plants to confirm that the behavior observed is truly correlated with actin distribution and dynamics.

### Functional relevance of stromules in the context of retrograde signaling

At the beginning of this study it was not clear whether stromule-nucleus associations are occurring by chance. This work has clearly established that in *A. thaliana* leaf model tissue a “stromule-promoting zone” exists. This could be seen as support for the hypothesis that stromules are intentionally formed as pathways for retrograde signals. However beside its clear existence, the functionality of this zone in the context of plastid-nucleus communication remains open. Through the characterization of unchallenged *A. thaliana* plastid position it was observed that there are typically two or more plastids with or without a stromule in direct “contact” (fluorescence signals overlap) with the nucleus of most cells (Figures 2B,C). If direct contact between the nucleus and plastid is a requirement for communication then it could be argued that plastids positioned at the nucleus should not need to form stromules, and yet this frequently occurs. Further, it was observed that approximately half of the epidermis cells (567 out of 1,213) studied in still images lacked stromules completely. Assuming stromules are truly important for communicating plastid “status” to the nucleus, the lack of stromules in unchallenged tissue may indicate that these cells do not require as much plastid-nucleus communication, but that this process is required for fast response in times of high stress. An alternative explanation is that stromules are not required for retrograde communication at all. The reliable association of plastids with the nucleus in every cell may be enough to ensure that plastid derived signals reach their destination. Interestingly, the number of plastids associated with the nucleus was only partially correlated with the cell size, meaning that although in larger cells the total number of plastids increased, the average number of nucleus associated plastids remained constant (Figure 2A). Given these results, one could speculate that only a few nuclear-associated plastids are sufficient to inform the nucleus about the condition of plastids throughout the cell.

As outlined earlier, time-lapse movies suggest that stromule formation within the “stromule promoting zone” appears to be the result of opposing movement of plastids and nuclei. Interestingly, as illustrated by Supplementary Movie 3 and Figure 8B, in the large majority of cases plastids will follow the nucleus when it moves over a significant distance. This gives the impression that the plastid bodies are “dragged” behind the nucleus by stromules. When the nucleus changes its direction, “returning” to the plastids, stromules shrink as the nucleus comes closer to the plastid bodies (Figures 8A,B). This type of coupling between plastid body movement, nucleus movement and stromule extension was also observed by Caplan et al. (2015) during hypersensitive response in *N. benthamiana*. The frequent observation of this behavior gives the impression that stromules may simply help keep plastids in contact with the nucleus, analogous to a retractable leash. Considering that in over 99% of all cells plastids are in direct contact with the nucleus, it seems that plastid body-nucleus associations are fundamental in the epidermis. Further the phenomenon of plastids clustering around the nucleus is frequently observed in plants under a wide variety of biotic challenges (Supplementary Table 1), emphasizing the importance of plastid body-nucleus position to stress response. These observations lead to the hypothesis that instead of/in addition to relaying signals to the nucleus, stromules act as anchors to maintain a certain plastid body-to-nucleus proximity. It has been suggested before that stromules could have a role maintaining plastid position within the cell (Hanson and Sattarzadeh, 2008), however this data provides the first support for a role in the context of coupling plastid and nuclear movement in unchallenged plant tissue.

This work does not exclude the possibility that signals, such as H_2_O_2_, travel through stromules toward the nucleus, as was demonstrated by Caplan et al. (2015). Fundamentally, stromules consist of stroma in which molecules (potentially including retrograde signals) can diffuse. However, as already mentioned, observations presented here suggest that in the upper epidermis of *A. thaliana* leaves stromules are not specifically extended from plastids residing further away from the nucleus to “reach out” for, and connect to it (see Figure 8C). Instead, the vast majority of stromule initiations within the stromule-promoting zone seem to be dependent on the relative position of the nucleus and the plastid, potentially to maintain plastid-nucleus contacts despite constant nuclear movement (see Figure 8D). Regardless of whether stromules have a significant role in retrograde signaling and/or plastid positioning the result is the same, plastids within 8 μm of the nucleus have stromules facing toward the nucleus. The key difference is that a role for stromules in retrograde signaling implies stromule growth would have to be guided toward the nucleus by an unknown mechanism. In contrast, a theory including a role for stromules acting as plastid anchors is already sufficient to explain stromule directionality and behavior.

## Conclusion

In recent years there has been a wealth of observations of stromules/plastid bodies associating with nuclei and much speculation as to the significance of such interactions. Most recently stromules have been implicated in retrograde signaling. However, very few stromule-nucleus observations have been quantified in any way, and none have characterized the position of stromule forming plastids within the context of the whole cell. Originally it was suspected that the result of quantifying the plastid/stromule-nucleus relationship would lead to support for one of two hypotheses, the first being that the stromule is indeed important for nuclear contact and directionally extends toward it, and the second being that stromule contacts are simply formed as a result of an overall inflation of stromule frequency, and are thus coincidental. Still images provided strong support for the former when they revealed that stromules are clearly forming near the nucleus and often appear to be “reaching” toward it, perhaps facilitating retrograde signal transfer. However time-lapse movies renewed doubt regarding a role for these structures in plastid-nuclear communication when they revealed that stromules observed were never seen growing in the direction of the nucleus. Surprisingly, the vast majority of stromules observed were extended as a result of the nucleus and plastid moving in opposing directions. This new evidence suggests that stromules may have a more indirect role in organelle communication, perhaps acting to maintain a certain number of plastid bodies at the nucleus to facilitate signal transfer between these two organelles.

## Author contributions

JE collected measurements and wrote the article. MK collected and analyzed data. MS collected data, analyzed data and supervised the writing of the manuscript.

### Conflict of interest statement

The authors declare that the research was conducted in the absence of any commercial or financial relationships that could be construed as a potential conflict of interest.
