# Supplementary material for: Plastid-Nucleus Distance Alters the Behavior of Stromules
Source: Front Plant Sci. 2017 Jul 6;8:1135. doi: 10.3389/fpls.2017.01135 (PMC5498514; doi:10.3389/fpls.2017.01135)
Supplement: Supplementary file 1 [file Table1.DOCX]

Supplementary Material

Plastid-nucleus distance alters the behavior of stromules

Jessica Lee Erickson, Matthias Kantek, Martin Hartmut Schattat*

* Correspondence: Dr. Martin Harmut Schattat: martin.schattat@pflanzenphys.uni-halle.de

**Table 1.** Literature survey of publications that mention stromule/plastid-nucleus relationships.

| **Organism/**  **Tissue** | **Condition** | **Observations** | **Speculated role of stromules** | **Citation** |
| --- | --- | --- | --- | --- |
| *Nicotiana benthamiana* - mature leaf epidermis | Transient p50 induced hypersensitive response in N-containing NRIP1-Cerulean plants | Stromules wrapped around nucleus, stromule tips in contact with nucleus, stromule associated with nuclear grooves, increase in stromules, stromule nuclear contact was correlated with enhanced hypersensitive response/programmed cell death | Stromules amplify HR-PCD defense signals to the nucleus, speculated role in retrograde signaling | Caplan et al., 2015^1^ |
| *Solanum tuberosum* - phelloderm | Carboxyfluorescein diacetate (CFDA) stained tissue | Plastids cluster around nuclei with short stromules pointing inward or longer stromule directed outward toward the cell periphery | Stromules signal physiological status of plastid to the nucleus or cell membrane | Borucki et al., 2015 |
| *Nicotiana benthamiana* - mature leaf lower epidermis | Stable ferredoxin NADP(H) oxidoreductase (FNR) transit peptide-EGFP plants infiltrated with *Agrobacterium tumefaciens* strain GV3101 (pMP90) or GV3101 overexpressing the bacterial *tzs* gene or direct infiltration with cytokinins | Plastids cluster around the nuclei exhibiting stromules facing both towards and away from the nucleus. Overexpression of trans-zeatin synthase led to the observation of plastid clusters around the nucleus with stromules radiating outward^3^ | No speculations about the function of stromules or plastids in this context | Erickson et al., 2014 |
| *Nicotiana benthamiana* - mature leaf epidermis | Transient *Abutilon* mosaic virus (AbMV) / cpHSC70-1 (*Arabidopsis thaliana*)- YFP (BiFC) | Stromules arose from plastids associated with nucleus, sometimes wrapping around the nucleus | Stromules could transfer macromolecules inter or intracellularly, with a proposed role in viral AbMV propagation | Krenz et al., 2010 and 2012 |
| *Arabidopsis thaliana* - rosette leaf mesophyll cells | High temperature treatment (35ºC) for 2 hr | Stromule membranes came into close contact with nuclear membranes | Stromules could have a role in signal transfer or metabolite trafficking | Holzinger 2007 |
| *Nicotiana tabacum*- callus cell | Stable plastid-targeted GFP | Plastids cluster around nuclei and long stromules radiate outward | Stromules may increase plastid surface area available for exchange/or facilitate close interactions required for the exchange of 'materials' between the plastid and the nucleus | Hanson and Sattarzadeh, 2008 |
| *Nicotiana tabacum*- seedling hypocotyl epidermis | Stable expression of triose phosphate 3-phosphoglycerate phosphate translocator (TPT) GFP | Plastids cluster around nuclei and emanate long stromules toward periphery | Stromule-nuclear contacts could play a role in signaling between nucleus and plastid^2^ | Natesan et al., 2005 |
| *Nicotiana tabacum*- petal epidermis | Stable overexpression of Rubisco small subunit RbcS-3A (*Pisum sativum*) CFP and SV40 large T-antigen nuclear localization signal mGFP4 | Plastids cluster around nuclei and stromules extended inward, some appear to contact the nucleus | Plastids and stromules within nuclear grooves may facilitate exchange of 'molecules and signals' between these organelles. Stromules extending toward the plasma membrane may facilitate interaction between nuclei/plastids and the plasma membrane | Kwok and Hanson, 2004 |
| *Nicotiana tabacum*-seedling hypocotyl epidermis, cotyledon petiole cells | Stable plastid targeted GFP grown in light or dark | Plastids densely clustered around the nuclei with long stromules extending toward periphery |  |  |
| *Nicotiana tabacum* - liquid-cultures suspension | Stable plastid targeted GFP cell in a suspension of NT1 medium |  |  |  |
| *Nicotiana tabacum* - hypocotyl epidermis | Stable overexpression of Rubisco small subunit RbcS-3A (*Pisum sativum*) CFP and SV40 large T-antigen nuclear localization signal mGFP4 | Perinuclear plastids were observed with stromules extending into nuclear envelope invaginations |  |  |
| *Nicotiana tabacum*- root | Stable overexpression of recA transit peptide GFP | In the meristematic zone most plastids clustered around what looks like nuclei with stromules extending toward periphery. The elongation, differentiation zone and mature root cells show slightly less clustering of plastids, mature root shows shorter stromules (note: stromule density is very high in all cells^3^) | Stromules could facilitate exchange of molecule between the nucleus and the plastid | Köhler and Hanson 2000 |
| *Nicotiana tabacum*- callus cell | Stable overexpression of recA transit peptide GFP, callus cells placed into NT1 medium | Most cells have the majority of plastids clustered around the nucleus with stromules extending toward periphery, stromules can be very long (40µm) and very dynamic |  |  |

^1^Caplan et al. (2015) quantified the number of stromule to nuclear associations, but not in the context of all the plastids in the cell

^2^Natesan et al., (2005) clearly states that the nature of the stromule-nucleus interaction needs to be characterized in more detail before real conclusions can be drawn

^3^Observations made during the examination of images in the publication

**References**

Borucki, W., Bederska, M., Sujkowska-Rybkowska, M. (2015). Visualisation of plastid outgrowths in potato (*Solanum tuberosum* L.) tubers by carboxyfluorescein diacetate staining. *Plant Cell Rep* 34, 853-860. doi: 10.1007/s00299-015-1748-2.

Caplan, J. L., Kumar, A. S., Park, E., Padmanabhan, M. S., Hoban, K., Modla, S., Cyzmmek, K., and Dinesh-Kumar, S. P. (2015). Chloroplast Stromules Function during Innate Immunity. *Dev. Cell* 34, 45–57. doi:10.1016/j.devcel.2015.05.011.

Erickson, J. L., Ziegler, J., Guevara, D., Abel, S., Klösgen, R. B., Mathur, J., Rothstein S. J., Schattat, M. H. (2014). Agrobacterium-derived cytokinin influences plastid morphology and starch accumulation in *Nicotiana benthamiana* during transient assays. *BMC Plant Biology* 14, 127-147. doi: 10.1186/1471-2229-14-127.

Hanson, M. R., and Sattarzadeh, A. (2008). Dynamic morphology of plastids and stromules in angiosperm plants. *Plant Cell Environ* 31, 646–657. doi:10.1111/j.1365-3040.2007.01768.x.

Holzinger, A., Wasteneys, G. O., and Lütz, C. (2007). Investigating Cytoskeletal Function in Chloroplast Protrusion Formation in the Arctic-Alpine Plant Oxyria digyna. *Plant Biology* 9, 400–410. doi:10.1055/s-2006-924727.

Köhler, R. H., and Hanson, M. R. (2000). Plastid tubules of higher plants are tissue-specific and developmentally regulated. *Journal of Cell Science* 113, 81–89.

Krenz, B., Jeske, H., and Kleinow, T. (2012). The induction of stromule formation by a plant DNA-virus in epidermal leaf tissues suggests a novel intra- and intercellular macromolecular trafficking route. *Frontiers in Plant Science* 3, 1–12. doi:10.3389/fpls.2012.00291.

Krenz, B., Windeisen, V., Wege, C., Jeske, H., and Kleinow, T. (2010). A plastid-targeted heat shock cognate 70kDa protein interacts with the Abutilon mosaic virus movement protein. *Virology* 401, 6–17. doi:10.1016/j.virol.2010.02.011.

Kwok, E. Y., and Hanson, M. R. (2004). Plastids and stromules interact with the nucleus and cell membrane in vascular plants. *Plant Cell Rep* 23, 188-195. doi: 10.1007/s00299-004-0824-9.

Natesan, S. K. A., Sullivan, J. A., and Gray, J. C. (2009). Myosin XI Is Required for Actin-Associated Movement of Plastid Stromules. *Molecular Plant* 2, 1262–1272. doi:10.1093/mp/ssp078.
