# Supplementary material for: Plastid-Nucleus Distance Alters the Behavior of Stromules
Source: Front Plant Sci. 2017 Jul 6;8:1135. doi: 10.3389/fpls.2017.01135 (PMC5498514; doi:10.3389/fpls.2017.01135)
Supplement: Supplementary file 5 [file DataSheet1.DOCX]

Supplementary Material

Plastid-nucleus distance alters the behavior of stromules

Jessica Lee Erickson, Matthias Kantek, Martin Hartmut Schattat*

* Correspondence: Dr. Martin Harmut Schattat: martin.schattat@pflanzenphys.uni-halle.de

**Supplemental Movie 1 – movie_01.mp4**

**Description**: Movie depicting the movement of the nucleus and plastids within a single epidermis cell. In this movie it is visible that plastids in close proximity to the nucleus show repeated stromule initiations and retractions and that plastids gain or loose this ability depending on whether they enter or leave the stromule-promoting zone. The nucleus starts out with 3 plastids within the (numbered 3, 4 and 5). During the movie two plastids move out of this zone and lose their ability to form stromules thereafter (3 and 4). Movement of the nucleus toward plastid 6 causes this plastid to enter the zone. After the plastid is in close proximity to the nucleus the plastid gains the ability to form stromules and also starts to follow the movement of the nucleus.

**Reference to figures**: This cell was used to create panel E in Figure 7; **Type of data**: maximum intensity projection along the z-axis of a 3D time series, frames are 3 minutes apart; **Replay rate**: 12 frames per second; **Channels**: green = eGFP fluorescence (FNR-eGFP), red = mCherry fluorescence (H2B-mCherry), blue = chlorophyll auto-fluorescence; **Labels**: solid white line represents cell outline, numbers top left = time stamp representing hours:minutes, lower right scale bar; **Tissue**: Upper epidermis of *pLSU4::pn* transgenic *A. thaliana.* Bright green plastids reside in the epidermis cell, larger plastids exhibiting a strong chlorophyll fluorescence reside in the palisade parenchyma. Due to the maximum intensity projection stromules from one tissue can appear to originate from plastids of the other (e.g. first few frames plastid 6).
