# Supplementary material for: Plastid-Nucleus Distance Alters the Behavior of Stromules
Source: Front Plant Sci. 2017 Jul 6;8:1135. doi: 10.3389/fpls.2017.01135 (PMC5498514; doi:10.3389/fpls.2017.01135)
Supplement: Supplementary file 6 [file DataSheet2.DOCX]

Supplementary Material

Plastid-nucleus distance alters the behavior of stromules

Jessica Lee Erickson, Matthias Kantek, Martin Hartmut Schattat*

* Correspondence: Dr. Martin Harmut Schattat: martin.schattat@pflanzenphys.uni-halle.de

**Supplemental Movie 2 - movie_02.avi**

**Description**: Movie depicting the correlation between nucleus movement and stromule formation. At the beginning of the movie none of the plastids show a stromule. During the movie the nucleus moves away from the plastids and simultaneously stromules form pointing towards the moving nucleus. Plastids remain more or less stationary. At 1h and 6 minutes the nucleus changes direction moving back towards and beyond the plastids. During this movement plastid stromules shorten, extend and continuously point towards the moving nucleus.

**Reference to figures**: This movie was used to create panel A in Figure 8; **Type of data**: maximum intensity projection along the z-axis of a 3D time series, frames are 3 minutes apart; **Replay rate**: 12 frames per second; **Channels**: green = eGFP fluorescence (FNR-eGFP), red = mcherry fluorescence (H2B-mCherry), blue = chlorophyll auto-fluorescence; **Labels**: numbers top left = time stamp representing hours:minutes, lower right scale bar; **Tissue**: Upper epidermis of *pLSU4::pn* transgenic *A. thaliana.* Bright green plastids reside in the epidermis cell, larger plastids exhibiting a strong chlorophyll fluorescence reside in the palisade parenchyma.
