# Supplementary material for: Plastid-Nucleus Distance Alters the Behavior of Stromules
Source: Front Plant Sci. 2017 Jul 6;8:1135. doi: 10.3389/fpls.2017.01135 (PMC5498514; doi:10.3389/fpls.2017.01135)
Supplement: Supplementary file 7 [file DataSheet3.DOCX]

Supplementary Material

Plastid-nucleus distance alters the behavior of stromules

Jessica Lee Erickson, Matthias Kantek, Martin Hartmut Schattat*

* Correspondence: Dr. Martin Harmut Schattat: martin.schattat@pflanzenphys.uni-halle.de

**Supplemental Movie 3 - movie_03.avi**

**Description**: Movie depicting the correlation between nucleus movement and stromule formation. Movie illustrates how a nucleus 'drags' plastids along when moving over longer distances. At the beginning of the movie none of the plastids show a stromule. During the movie the nucleus moves away from the plastids and simultaneously stromules form pointing towards the moving nucleus. When the distance increases to a certain maximum all plastids, except the furthest to the right follow the nucleus with the same speed. The plastid on the far right remains stationary and effectively leaves the stromule-promoting zone, consequently losing its stromule. Stromules of the residual three nuclear-associated plastids show a change in direction and elongation behaviour according to the change in the nucleus movement.

**Reference to figures**: This movie was used to create panel B in Figure 8; **Type of data**: maximum intensity projection along the z-axis of a 3D time series, frames are 3 minutes apart; **Replay rate**: 12 frames per second; **Channels**: green = eGFP fluorescence (FNR-eGFP), red = mcherry fluorescence (H2B-mcherry), blue = chlorophyll auto-fluorescence; **Labels**: numbers top left = time stamp representing hours:minutes, lower right scale bar; **Tissue**: Upper epidermis of *pLSU4::pn* transgenic *A. thaliana.* Bright green plastids reside in the epidermis cell, larger plastids exhibiting a strong chlorophyll fluorescence reside in the palisade parenchyma.
